# Auto-Combustion of Corn Straw: Production and Characterization of Corn Straw Ash (CSA) for Its Use in Portland Cement Mortars

**DOI:** 10.3390/ma17174374

**Published:** 2024-09-04

**Authors:** Jordi Payá, Alejandro Escalera, María Victoria Borrachero, Josefa Roselló, José Monzó, Lourdes Soriano

**Affiliations:** 1 ICITECH—Institute of Concrete Science and Technology, Universitat Politècnica de València, Av. dels Tarongers, 4N, 46022 València, Spain; alescru@gmail.com (A.E.); vborrachero@cst.upv.es (M.V.B.); jmmonzo@cst.upv.es (J.M.); lousomar@upvnet.upv.es (L.S.); 2 Department of Agroforestry Ecosystems, Universitat Politècnica de València (UPV), 46022 València, Spain; jrosello@upvnet.upv.es

**Keywords:** biomass, auto-combustion, thermogravimetry, scanning electron microscopy, mortar, compressive strength, pozzolan, supplementary cementing material

## Abstract

Agricultural waste availability implies the possibility of recovering energy as biomass. The collateral effect is the production of ashes that, in some cases, have the potential to be reused in the manufacture of cement, mortar, and concrete. This article presents the study of the auto-combustion (unlike all previous studies) of corn (maize) straw (stems and leaves). The auto-combustion temperature was monitored, and the obtained corn straw ash (CSA) was characterized by means of X-ray fluorescence, X-ray diffraction, thermogravimetry, and scanning electron microscopy. Finally, the behavior of ground CSA was analyzed in both the fresh state by measurement of workability on the spreading table and the hardened state by compressive strength measurement on mortars in which 10% of ordinary Portland cement (OPC) was replaced with CSA. These values were compared to both a control mortar (OPC) and a mortar in which OPC was partially replaced with 10% limestone filler. Ashes showed adequate pozzolanic reactivity because, at 90 curing days, the compressive strength of the mortars with 10% replacement of OPC with CSA was practically equal (98% of the strength) to the control mortar without pozzolan replacement. The auto-combustion of biomass is a process that can be easily available, and the results on pozzolanic reactivity of CSA are satisfactory. The auto-combustion could be used by low-income communities to reduce Portland cement clinker use and to recover waste.

## 1. Introduction

The need to develop new binders with lower or no clinker content and a low carbon footprint is a reality for today’s world if we intend to fight climate change [1,2]. Supplementary cementitious materials (SCMs) availability may be limited, and new SCMs must become part of these new types of cement. For example, the closure of coal-fired power plants in many countries and less coal use significantly lower fly ash availability. The production of these fly ashes is reported to be of the order of 330 Mt/year [3], which represents a fly ash/cement ratio of 0.08 (if we accept the estimation of 4100 MT/year world cement production reported in 2023 [4]). In this way, the fly ash/cement ratio is barely 0.08 t/t. Another waste material that is widely used in cement manufacturing is blast furnace slag (BFS). The demand for ferrous products has continued to increase in recent years, and in 2022, the demand for steel was 1790 Mt/year, with a projection of 2030 Mt/year by 2030 [5]. BFS production in 2022 was 408 Mt, with a projection of 497 Mt by 2035 [6]. However, the slag/cement ratio is only 0.10 t/t. The valorization of silica fume (SF) from the silicon and ferrosilicon industry is very interesting and effective due to the high pozzolanic reactivity of SF. However, its annual production is estimated at about 3 Mt because 1 t of SF is produced for every 3 t of manufactured elemental silicon [7] and given that silicon production is 9 Mt/year [8].

**Figure 1:**
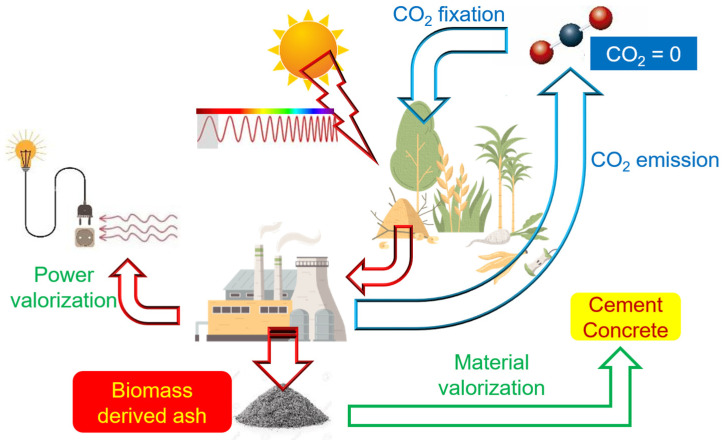
Scheme of the energy recovery of biomass and the material recovery of biomass ashes in cement/concrete.

Other SCM alternatives are natural materials, such as volcanic rocks, diatomaceous earth, or clays. However, there is a set of interesting resources from the point of view of binding materials in construction: agricultural/forestry waste [9]. One of the most widespread and highest-producing crops is *Zea mays* L. (maize or corn)., world corn production is approximately estimated at 1137 Mt (data from 2019) [10]. One part of the biomass residue is the corn cob, which remains after removing grains from spikes. Another important waste is the plant itself (stems, sheat leaves, leaves, roots). The corn cob and the rest of the plant are residual materials that can be energetically valorized by generating energy from oxidation by organic matter combustion. As the CO_2_ emitted during combustion has been previously fixed by the plant, this process is practically neutral in carbon footprint terms (Figure 1). Energy recovery can be carried out by combustion, which results in biomass ash that may have suitable properties to be used as an SCM.

Table 1 summarizes the theoretical amount of ash produced from selected crops. These crops are the world’s most popular ones: rice, wheat, sugarcane, and corn. The biomass with the highest proportion of ash is rice husk (22%), while rice straw, maize straw, and wheat straw fall within the 10–15% range. Taking into account the proportion of residue per food unit and the proportion of ash in the biomass, the theoretical quantities of ash that can be produced worldwide have been calculated. Of the seven biomasses, that which could produce the biggest amount of ash is corn straw, with 154 Mt/year. However, the corn cob only has an ash generation potential of 4 Mt/year.

Many reports [11] have been published on the use of corn cob ash (CCA) in the formulation of cements and concrete. This ash (CCA) has shown high pozzolanic reactivity due to the presence of amorphous silica, while the sum of oxides SiO_2_, Al_2_O_3_, and Fe_2_O_3_ exceeds 70%, which is one of the conditions of standard ASTM C618 [12] to be considered pozzolanic material.

There are fewer studies on corn straw ash (CSA) [13]. De Lima and Cordeiro [14] published the physico-chemical properties of ash obtained at a temperature of 600–650 °C and observed the high amorphicity of ash in mineralogy terms. In addition, 62.5% of SiO_2_ and a high percentage of K_2_O (17.2%) were determined. They observed how the contribution to the mechanical compressive strength (Rc) of mortars with 10%, 20%, and 30% cement replacements allowed slightly higher Rc values to be obtained than in the control mortar. When CSA is washed with acid, silica content increases (94.2%), all of which is amorphous: the Rc of mortars was between 10% and 50% higher than in the control after 28 curing days. Qi et al. [15] studied the effect of calcination temperature (500, 700, and 850 °C) on the pozzolanic reactivity of corn straw stem (stalk) ash (MSSA). In this case, the percentage of silica was between 31% and 38%, while K_2_O fell within the 21–28% range. Large amounts of calcium, magnesium, and chlorides were also found. The most abundant mineral was sylvite (KCl). However, after washing ashes with water, calcite and quartz were the majority minerals, along with amorphous silica. The authors concluded that the sample with the highest reactivity was that generated at 500 °C. Raheem et al. [16] studied several CSA samples obtained by burning stalks in an open steel container. In this case, the average SiO_2_ content was high (64.26%), unlike that found in [15]. The following contents were observed: 9.45% K_2_O, 6.23% CaO and 5.28% Al_2_O_3_. The authors concluded that the 10% replacement of cement with CSA was the optimum one for interlocking paving stone in compressive strength terms, and the 10–25% replacement range was optimum regarding abrasion. A study was also found about corn leaf ash (CSLA) obtained at three different combustion temperatures: 500, 700 and 850 °C [17]. The SiO_2_ content fell within the 67–74% range, and the sum of SiO_2_ + Al_2_O_3_ + Fe_2_O_3_ ranged from 70% to 78%. Pozzolanic reactivity was evaluated by taking electrical conductivity measurements, which revealed that the sample obtained at 500 °C was the most reactive one. The reactivity of the sample obtained at 850 °C had decreased despite having more SiO_2_ because silica crystallization occurred in the form of cristobalite. The reaction of CSLA with Ca(OH)_2_ solution led to the formation of gismondine and calcium silicate hydrate (C-S-H).

Previous reports have indicated studies into CSA obtained under controlled laboratory conditions. Due to the availability of this biomass in low-income environments (communities in developing countries), the aim is to study the ash obtained by the auto-combustion of the biomass to evaluate the properties of the CSA to be used in PC matrices.

## 2. Materials and Methods

**Figure 2:**
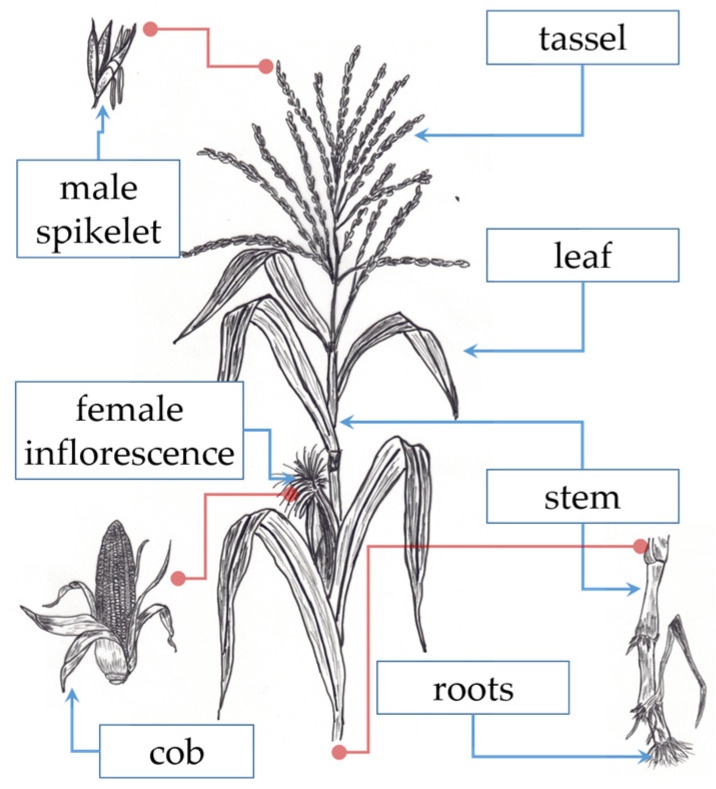
Scheme of the main maize plant parts. The red lines correspond to a magnification of the plant part.

The corn straw (CS) samples were acquired from two commercial suppliers (from Pabellón de Arteaga, Aguascalientes, Mexico). The “rastrojo” term is used in Mexico to define this type of biomass. CS contains mainly leaves, leaf sheaths, and stems of the plant, but also other parts [18], such as tassels (male inflorescences), roots, silks (stigmas), leaf blades, and corn cobs. Figure 2 represents the most important maize plant parts.

**Figure 3:**
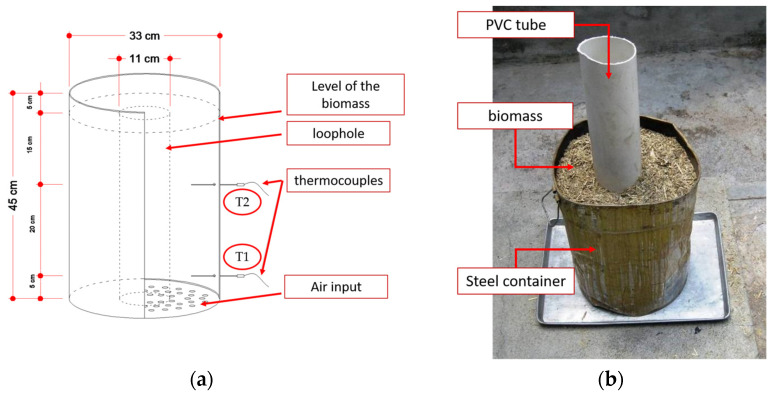
Auto-combustion of corn straw: (**a**) scheme and dimensions of the steel container; (**b**) preparation before combustion.

The two commercial “rastrojo” samples were named R1 and R2. These biomass samples went from 1 mm to 3 cm and came in the form of dry chips. The auto-combustion of R1 and R2 was carried out in a cylindrical steel container. Figure 3a shows the furnace dimensions, and Figure 3b outlines the layout of the material inside the oven. The biomass was compacted by hand and a PVC pipe was placed so that, after removal, a chimney (loophole) was obtained for rapid and effective biomass combustion. Two thermocouples (T1 at the bottom and T2 at the cylinder’s mid-height) were used for monitoring changes in temperature.

**Figure 4:**
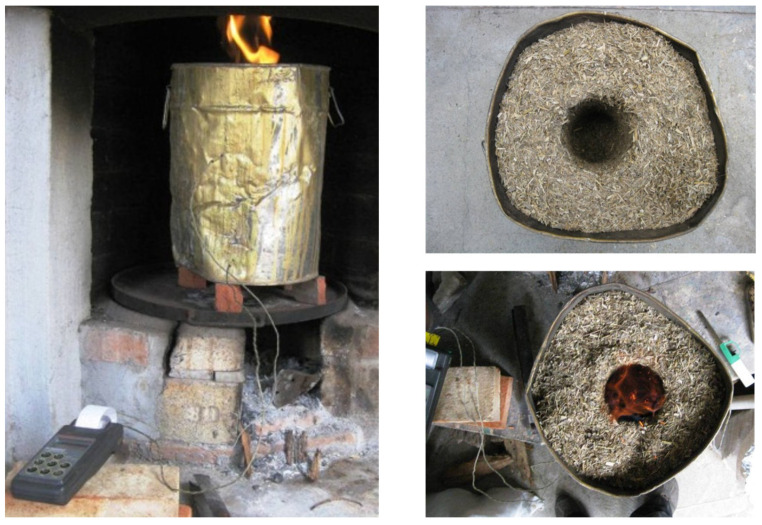
Some pictures of the combustion process.

Figure 4 shows the combustion process. After combustion and cooling (1 day) the resulting ashes, ashes were stored in airtight containers. The ashes obtained from R1 and R2 were CSA-R1 and CSA-R2, respectively.

Two other additional samples were collected (cut from fresh plants) from a crop field in Aguascalientes City (Mexico) and corresponded to the leaf and stem parts (samples MX-L and MX-S, respectively).

A furnace (model RHF 15/3 Carbolite, Hope Valley, UK) was used to determine the loss on ignition (LOI) of ashes and to prepare the selected samples from corn leaves. The thermogravimetric (TG) characterization of the biomass samples (R1 and R2) and ashes (CSA-R1 and CSA-R2) was carried out in a TGA-850 (Mettler-Toledo, Metller-Toledo S.A.E, Cornellà del Llobregat, Spain) using 70 µL alumina crucibles at a heating rate of 20 °C/min and an air gas flow of 75 mL/min. The chemical composition of cement and CSAs was determined by X-ray fluorescence with Philips MagiXPRO equipment (Philips Analytical, Almelo, The Netherlands). The mineralogical composition of raw materials was determined by XRD (Philips diffractometer PW1710, Philips Analytical, Almelo, The Netherlands) with Cu Kα radiation, 40 kV and 20 mA, from 10 to 80° (2Θ). SEM-EDX observations were performed by a JEOL JSM-6300 model (Hertfordshire, UK). Samples were covered with carbon.

To prepare mortars, CEM I 52-5R (OPC, Lafarge-Holcim, Puerto de Sagunto, Spain) and siliceous sand (Caolines Lapiedra, Liria, Spain) were used. The 10% replacement by mass of OPC with CSA-RM was carried out to have a sufficient quantity of ash to prepare the mortars for the tests at 28, 56, and 90 days of curing. This sample was prepared by mixing CSA-R1 and CSA-R2 in a 50/50 proportion (“M” means a mixture of ashes R1 and R2). CSA-RM was ground in a Gabrielli Mill-2 equipment (Gabrielli Technology, Calenzano, Italy) using 2-cm diameter alumina balls, 200 g of ash, and 20-min grinding. The particle size distribution of CSA-RM before and after grinding was determined by Mastersizer 2000 equipment (Malvern Instruments S.L., Malvern, UK) with samples suspended in deionized water for measurements (mean particle diameter d_m_, and percentiles d(0,1), d(0.5), and d(0.9)). Mortars were prepared with a cement/sand ratio of 1:3 and a water/cement ratio of 0.5 according to standard EN-196-1 [19]. For comparison purposes, limestone filler (supplied by Cementval, Puerto de Sagunto, Spain) was also used to replace 10% of CEM I 52.5R. Fresh mortars were characterized by workability (flow table spread; FTS) according to UNE-EN 413-2:2017 [20]. The specimens in the mold were stored in a moist atmosphere and, after 24 h, were demolded and stored in a saturated lime solution. Specimens (4 × 4 × 16 cm) were tested in the flexural (3 values) and compressive (6 values) modes according to [19].

## 3. Results and Discussion

### 3.1. Characterization of Corn Straws

**Figure 5:**
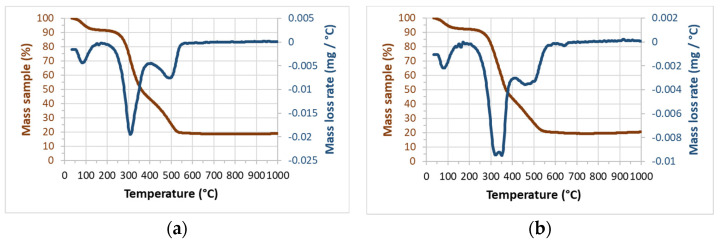
TG (red) and DTG (blue) curves for (**a**) R1 and (**b**) R2.

The finely ground R1 and R2 samples were dried in an oven at 105 °C for 24 h to determine the ash, volatiles, and non-volatile organic contents by thermogravimetry (air atmosphere). Figure 5 shows the TG and DTG curves for R1 and R2. They were very similar, with the total mass loss within the 79–82% range (see Table 2). The mass loss within the 100–120 °C range was attributed to moisture and adsorbed water. For the 250–400 °C range, mass loss was attributed to the volatilization of organic compounds. For the 400–600 °C range, mass loss was related to the combustion of non-volatile organic matter. The mass loss values are summarized in Table 2. The ash content for these samples came close to 20% because no mass loss occurs after 600 °C.

### 3.2. Auto-Combustion of Corn Straws

**Figure 6:**
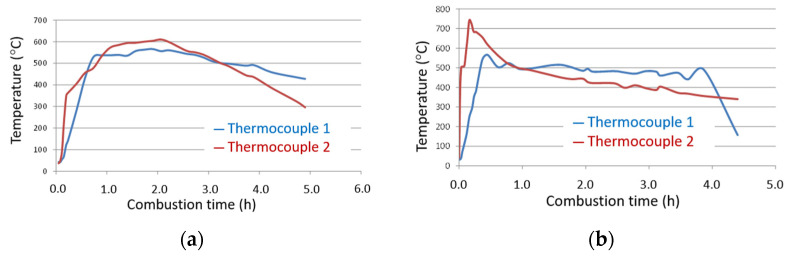
Monitoring the combustion temperature for: (**a**) R1; (**b**) R2.

The auto-combustion temperature in the furnace was monitored by two thermocouples. Figure 6 shows the evolution of the temperatures obtained for both samples R1 and R2. At both the monitored points (see Figure 3) and for both samples, the maximum temperature was reached between approximately 0.25 and 3 h. The maximum temperature was between 550 and 625 °C for sample R1 and between 575 and 750 °C for sample R2. The obtained amount of ash as a percentage was 14.85% for R1 and 12.02% for R2. These values were lower than those observed by thermogravimetry (Section 3.1) because biomasses were placed inside the furnace with no prior drying (the biomass samples were tested as received). These reached temperatures in the furnace that were high enough for complete volatile organic fraction volatilization and to oxidize part of the non-volatile part. So, the unburned residue was expected to be relatively low. In fact, the LoI determined for ashes was 6.54% for the ash from R1 and 3.20% for the ash from R2. These data show the marked effectiveness of the auto-combustion process.

### 3.3. Characterization of Corn Straw Ashes (CSA)

**Figure 7:**
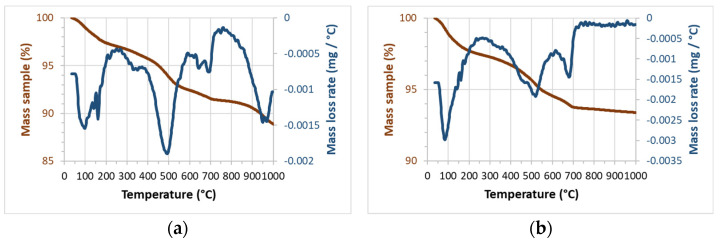
TG (in red) and DTG (in blue) curves for (**a**) CSA-R1 and (**b**) CSA-R2.

The ashes obtained from the auto-combustion of R1 and R2 were named CSA-R1 and CSA-R2. They were characterized by thermogravimetry, XRF, XRD, and SEM-EDS. The thermogravimetry study (TG and DTG curves) is shown in Figure 7. Both ash samples showed mass loss within the 35–200 °C range due to the moisture of the ash. A peak centered at about 500 °C due to the oxidation of the remaining organic matter. An additional peak occurred at almost 700 °C, which was attributed to the presence of carbonates (probably part of alkaline oxides, CaO, and K_2_O, uptake CO_2_ from the atmosphere, and conversion into carbonates took place). Finally, minor mass loss was observed at a temperature above 850 °C (peak centered at 960–980 °C), which was attributed to chloride volatilization. This peak was not observed for the CSA-R2 sample, which suggests that the percentage of chlorides in that ash was not high. Table 3 summarizes the mass losses for each temperature range.

**Figure 8:**
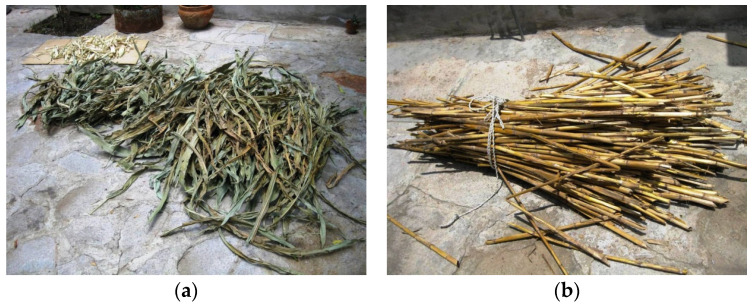
Selected biomasses: (**a**) corn leaves; (**b**) corn stems.

The chemical composition of the ashes was determined by XRF. The corresponding data are summarized in Table 4. The SiO_2_ content was 58.68% for CSA-R1 and 69.62% for CAS-R2. These values are similar to those reported for CSA (from stem + leaf) [14], corn stem ash [16], and CSLA [17] but are very different from those reported by [15] (See Table 5). This behavior highlights the utmost importance of the origin of the biomass. In Table 4, the ashes obtained from stems (CSA-MX-S) and leaves (CSA-MX-L) in the described furnace (Figure 4) are also chemically characterized; pictures of the materials are shown in Figure 8. Ashes from leaves have a higher SiO_2_ proportion than ashes from stems, and, inversely, K_2_O content is higher for ashes from stems. This means that SiO_2_ accumulation is produced mainly in corn leaves [21]. It was also noted that P_2_O_5_ content was higher for these biomass ashes than for those observed for the CAS-R1, CSA-R2, and biomass ashes summarized in Table 5. This suggests a strong influence on chemical composition depending on the biomass state (dry in the crop field or fresh). The chloride content was different: in CSA-R1, it was 4.28%, and it was 0.63 for CSA-R2. This is the reason the TG curve for the first ash presented significant mass loss at a temperature higher than 850 °C (Figure 7a). This behavior has also been reported [15]: ash samples obtained at 850 °C present 3.09% of chloride, whereas for the sample prepared at 500 °C the percentage is significantly higher (5.22%): chlorides (probably as KCl) are volatilized at high temperature. The relatively high percentages of both Al_2_O_3_ and Fe_2_O_3_ in CSA-R1 and CSA-R2 were attributed to the soil contamination of the starting biomass.

**Figure 9:**
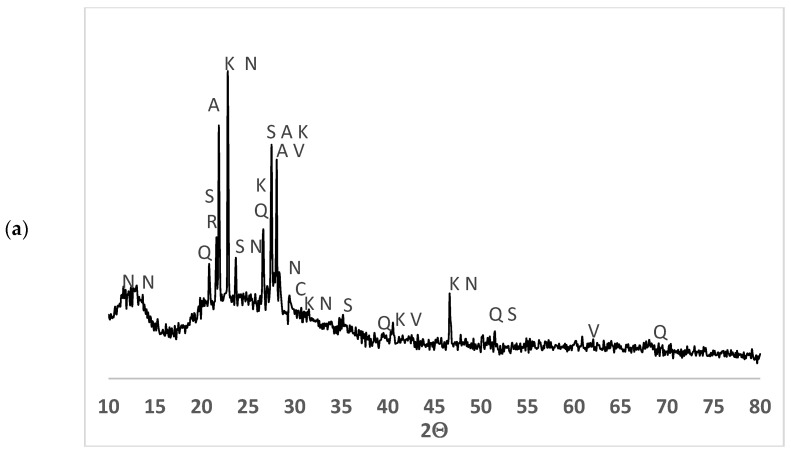

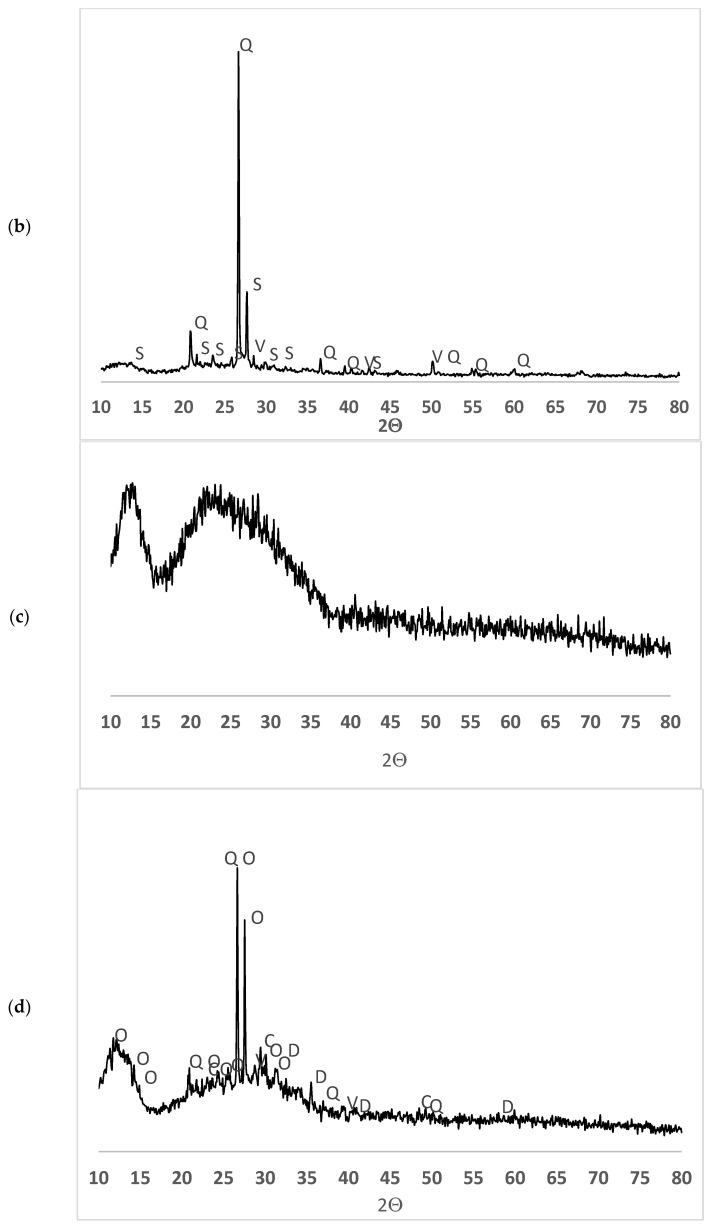
XRD diffractograms for biomass ashes: (**a**) CSA-R1; (**b**) CSA-R2; (**c**) CSA-MX-L; (**d**) CSA-MX-S. Legend: C = calcite (pdfcard 050586), V = sylvite (pdfcard 411476), Q = quartz (pdfcard 331161), A = anorthite (pdfcard 411486), S = sanidine (pdfcard 191227), R = cristobalite (pdfcard 391425), N = sodium calcium sulfate (pdfcard 341238), K = calcium potassium phosphate (pdfcard 391408), O = orthoclase (pdfcard 711540), D = diopside (pdfcard 190239).

Ashes were characterized by XRD. The diffractograms for CSA-R1 and CSA-R2 are depicted in Figure 9a and Figure 9b, respectively. CSA-R1 presented some minerals (mainly calcite, sylvite, quartz, anorthite, sanidine, cristobalite, calcium-sodium sulfate, and calcium-sodium phosphate). Some of these minerals are related to soil contamination (calcite, quartz, anorthite, and sanidine), and others were produced during biomass combustion. The presence of cristobalite suggests that part of the amorphous silica (present in the biomass) was converted into a crystalline phase. This has also been reported by Qi et al. [17]: the formation of cristobalite for ashes obtained at 850 °C. CSA-R2 presents quartz (the main crystalline phase, with significantly higher signal intensity than in the CSA-R1 XRD diffractogram), sylvite, and sanidine. For both ashes (CSA-R1 and CSA-R2), there was a deviation from the baseline within the 2Θ range of 20°–30°, which corresponded to amorphous silica. The plant accumulates silica [22,23] in cellular structures in the form of silica gel (SiO_2_·nH_2_O). The ash obtained from corn leaves CSA-MX-L (Figure 9c) did not show any crystallized material because leaves were meticulously washed with deionized water before auto-combustion (soil contamination was removed). The ash from washed stems CSA-MX-S (Figure 9d) presented small amounts of quartz, calcite, diopside, and orthoclase (soil contamination was not completely removed) and sylvite. An important amorphous phase in both cases occurred, which is the key parameter for yielding an excellent pozzolanic reaction.

**Figure 10:**
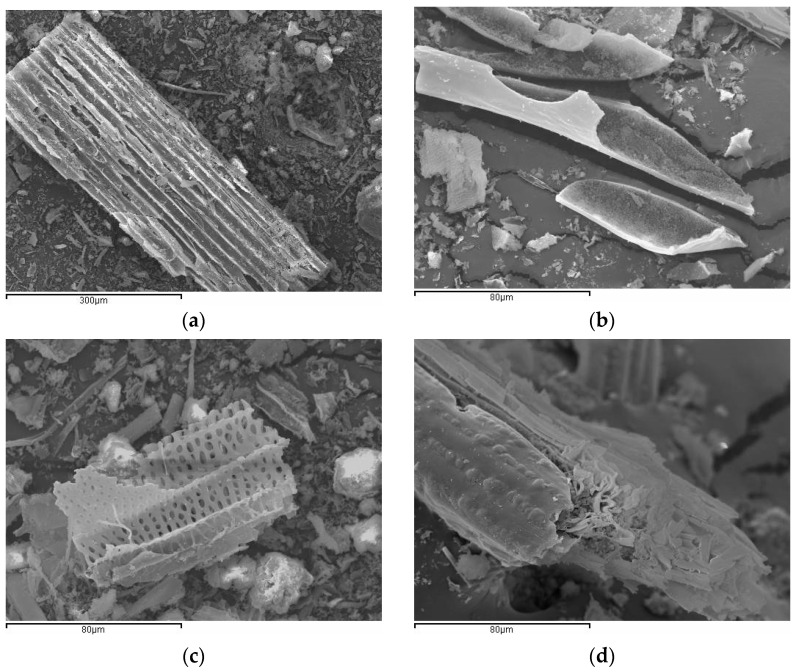
SEM micrographs of ashes: (**a**,**b**) CSA-R1; (**c**,**d**) CSA-R2.

**Figure 11:**
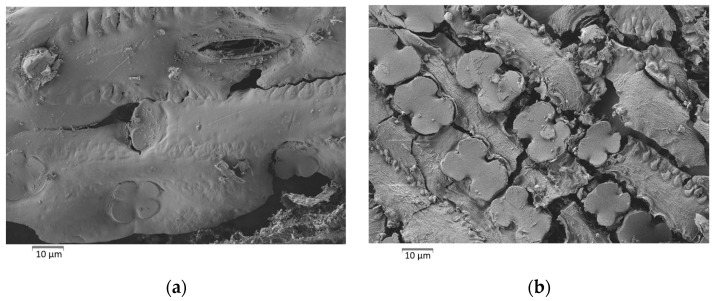
SEM micrographs of the corn leaf calcined at 450 °C: (**a**) leaf adaxial side; (**b**) leaf abaxial side.

Ash particle morphology was studied by SEM. Figure 10 shows some micrographs of the obtained ashes. After combustion, the original cellular structure remained for some particles. Skeletons remained after calcination because of the presence of a significant percentage of silica reported for bamboo leaf and sugarcane leaf ashes [14,17,23]. The size of many particles was bigger than 100 µm, which denotes that grinding before use in cementing systems is required. Additionally, particles presented many pores, which favors water adsorption. Another SEM study was carried out on the washed leaf samples obtained in the furnace at 450 °C in an oxidizing atmosphere: under these conditions, a major part of volatile organics was removed, and the silica/carbon skeleton remained. Figure 11 shows two micrographs of the adaxial (Figure 11a) and abaxial (Figure 11b) sides of the calcined leaf. In both micrographs, tetralobated (cross-shaped type) phytoliths are observed, which have 90–94% of SiO_2_ and 3–6% of K_2_O. The greatest phytoliths abundance is observed on the leaf abaxial side, which is very common in this type of botanical species [24,25]. This was why the CSA-MX-L sample had higher SiO_2_ than the CSA-MX-S sample. If the aim is to obtain ash with a higher proportion of silica (more reactive ash from a pozzolanic point of view), it would be worthwhile separating leaves. In this way, the ash from stems (CSA-MX-S) would be yielded with a high proportion of potassium, which could be used as fertilizer.

### 3.4. Portland Cement Systems with CSA

**Figure 12:**
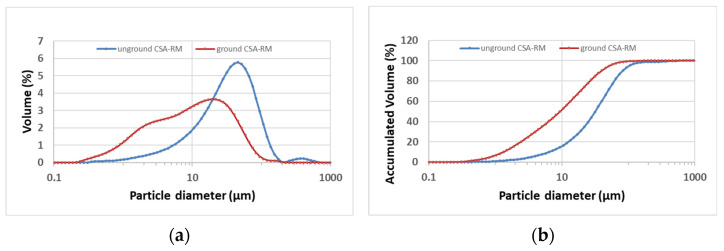
Particle size distribution of CSA-RM: (**a**) particle distribution curves; (**b**) accumulated volume curves (red curves for ground ash; blue curves for unground ash).

CSA-R1 and CSA-R2 were blended (50% of each) and ground. Grinding was necessary to reduce the water demand of porous ash particles. The particle size distributions before and after grinding are shown in Figure 12. The mean particle diameter of the ground sample was d_m_ =17.50 µm, and d(0.1) = 1.50, d(0.5) = 10.63 and d(0.9) = 43.70 µm. For the unground sample, they were d_m_ = 45.44, d(0.1) = 7.36, d(0.5) = 36.11 and d(0.9) = 91.53 µm. The ground material was in the appropriate state for mixing with OPC.

Mortars were prepared by replacing 10% of OPC with CSA-RM. A control mortar (without replacement) was prepared for comparison purposes. Fresh mortar workability was assessed by measuring FTS. The FTS for the control mortar was 151 mm, and it was 167 mm for the 10%-CSA-RM mortar. This means that grinding was very effective in terms of water adsorption in ash particles. The mortar prismatic specimens were cured under lime-saturated water for 28, 56, and 90 days. The mortar with 10% replacement with limestone filler was prepared to quantify the pozzolanic contribution of ash. Table 6 summarizes mortars’ flexural and compressive strengths.

On the one hand, the flexural strength development for the 10%-CSA-RM mortar was slightly lower than that for the control mortar and the 10%-filler mortar. On the other hand, the compressive strength of 10%-CSA-RM was also lower than that for the control mortar but was higher than that for the 10%-filler mortar. This implies the contribution of the pozzolanic reaction to the compressive strength development in the 10%-CSA-RM mortar. However, this contribution was not enough to reach the strength obtained for the control mortar, and the Strength Activity Index (SAI) equaled 0.92 for 28 and 56 curing days and 0.98 for 90 curing days. Despite the presence of a high percentage of SiO_2_ in ash (≈60%), the contribution of the pozzolanic reaction was limited: the presence of potassium salts probably affected the hydration process. De Lima et al. [14] and Raheen et al. [16] also reported similar or slightly higher strengths for the 10%-ash replaced systems to their corresponding control mortars. Witzleben [26] reported the negative influence of potassium hydroxide on the hydration and strength development of PC: the addition of 0.56% of KOH slightly reduced compressive strength at 2 curing days (from 39 to 35 MPa) but led to a marked reduction at 28 curing days (from 78 to 60 MPa). The presence of potassium in CSA-RM was responsible for the limited contribution to strength development.

## 4. Conclusions

Corn cultivation is the one that presents the greatest potential in terms of production of pozzolanic material from biomass, and it is estimated that the annual amount of ash could be more than 150 MT. The ash obtained from corn straw presents silica content close to 60% by mass and is a good candidate to play a pozzolanic role in OPC blends. The auto-combustion of this biomass produces corn straw ash (CSA), where silica is maintained in the amorphous phase because the temperature does not reach 750 °C. The total mass loss during the auto-combustion was close to 80%. The silica in CSA shows a certain reactivity level and is appropriate for blending with cement in mortars and concrete. XRD studies have shown that some soil impurities are found along with silica and these impurities can be easily removed by washing with water. CSA must be ground before being used to reduce water absorption and increase reactivity. SEM studies on ash particle morphology have shown that after combustion, the original cellular structure remains, and the resulting skeletons are composed of silica. The grinding reduced the particle size to a mean diameter close to 17.5 µm. Ashes showed adequate pozzolanic reactivity because, at 28 and 90 curing days, the compressive strengths of the mortars with 10% replacement of OPC with CSA were 92% and 98%, respectively, of the control mortar (without replacement). Ash contains a high fraction of potassium, which negatively affects the mechanical development of the mortar. Separation of leaves and stems would be an interesting approach to offer the best destination for ashes: from leaves to produce pozzolanic material (rich in amorphous SiO_2_) and from stems (ash rich in K_2_O) to produce fertilizers. This proposed auto-combustion of corn straw could be used by low-income communities to reduce Portland cement clinker use, fertilize crops, and recover waste.

Future studies should focus on the evaluation of the pozzolanic reactivity of water-washed CSA and on improving this reactivity by removing soluble potassium. On the other hand, the durability of mortars and concretes in aggressive environments should be tested.

## Figures and Tables

**Table 1:** Crops, biomasses, and ashes from the biomass for selected foods.

Crop	World Production of the Food (Mt/Year)	Biomass Name	Waste Biomass Proportion per Food Unit (%)	Ashes in the Biomass (%)	Theoretically Produced Ashes (Mt/Year)
Rice	500	Rice husk	20	22	22
		Rice straw	115	15	86
Wheat	780	Wheat straw	67	10	52
Corn	1200	Corn cob	15	2.2	4
		Maize straw	107	12	154
Sugarcane	1860	Sugarcane bagasse	20	2.5	9
		Sugarcane straw	150	5	140

**Table 2:** Thermogravimetric data (mass loss percentages within the selected temperature ranges) for R1 and R2.

Sample	35–200 °C	200–400 °C	400–600 °C	Total Mass Loss
R1	8.77	49.92	22.26	81.09
R2	7.95	49.50	22.40	79.52

**Table 3:** Thermogravimetric data (mass loss percentages within the selected temperature ranges) for CSA-R1 and CSA-R2.

Sample	35–200 °C	200–600 °C	600–1000 °C
CSA-R1	2.74	4.83	10.93
CSA-R2	2.63	4.96	11.13

**Table 4:** XRF chemical composition of the ashes obtained from the commercial “rastrojo” (CSA-R1 and CSA-R2) and the ashes obtained from leaves (CSA-MX-L) and stems (CSA-MX-S).

	CSA-R1	CSA-R2	CSA-MX-L	CSA-MX-S
Oxide	%	%	%	%
Na_2_O	0.68	0.63	0.00	0.55
MgO	2.83	2.49	3.67	3.48
Al_2_O_3_	5.50	7.60	1.33	2.26
SiO_2_	58.68	69.62	52.92	26.54
P_2_O_5_	2.10	2.52	8.06	9.43
SO_3_	1.74	0.89	1.83	1.88
Cl	4.28	0.63	2.52	0.68
K_2_O	15.41	7.83	21.47	40.14
CaO	4.96	4.81	7.61	12.25
Fe_2_O_3_	2.94	2.34	0.34	1.47

**Table 5:** XRF chemical composition of the ashes obtained and reported by other authors.

Reference	[13] ^a^	[14] ^b^	[15] ^c^	[16] ^d^
Plant Part	Straw (Stem + Leaf)	Stem	Stem	Leaf
Oxide	%	%	%	%
Na_2_O	nd	0.3	1.59	0.4
MgO	nd	5.73	3.89	3.68
Al_2_O_3_	nd	1.15	5.28	2.15
SiO_2_	62.5	31.34	64.26	67.02
P_2_O_5_	3	1.97	nd	0.94
SO_3_	2.6	0	3.43	2.42
Cl	nd	5.22	nd	0.39
K_2_O	17.2	21.44	9.45	5.24
CaO	8.7	4.27	6.23	6.78
Fe_2_O_3_	0.9	0.49	2.33	1.01

nd: not determined. ^a^: Biomass in nature, calcined at 600 °C; ^b^: sample obtained at 500 °C; ^c^: average of 4 samples, calcination temperature not reported; ^d^: calcination at 500 °C.

**Table 6:** Flexural and compressive strengths of the mortars cured at 28, 56, and 90 days.

Curing Age (Days)	Flexural Strength (MPa)			Compressive Strength (MPa)		
	Control	10%-CSA-RM	10%-Filler	Control	10%-CSA-RM	10%-Filler
28	6.75 ± 0.52	5.87 ± 0.32	6.51 ± 0.20	56.5 ± 1.6	52.1 ± 1.6	49.8 ± 2.4
56	6.65 ± 0.05	5.98 ± 0.41	6.35 ± 0.43	61.3 ± 1.7	56.4 ± 1.9	54.3 ± 2.4
90	7.70 ± 0.50	6.16 ± 0.28	7.16 ± 0.22	59.2 ± 2.1	57.8 ± 2.9	53.1 ±1.4

## Data Availability

Data will be made available upon request.
